# Supplementary material for: Factors associated with adolescent pregnancy among Chepang women and their health-seeking behavior in Ichchhakamana rural municipality of Chitwan district
Source: PLoS One. 2024 Mar 28;19(3):e0301261. doi: 10.1371/journal.pone.0301261 (PMC10977708; doi:10.1371/journal.pone.0301261)
Supplement: S1 Table — (DOCX) [file pone.0301261.s001.docx]

**S1 table. Variables of the study.**

| **SN** | **Variable Name** | **Definition** |
| --- | --- | --- |
| **Dependent variable** | | |
| 1 | Adolescent pregnancy | Any woman aged 15-20 years who had a birth within the last 1 year or woman aged 15-19 years who was pregnant during the time of the study |
| **Independent variables** | | |
| 2 | Age | Participant’s age at the time of interview |
| 3 | Religion | Religion that the participant follows |
| 4 | Education | Level of education that the participant has completed |
| 5 | Occupation | Type of job that the participant associates with |
| 6 | Family type | The kind of family that she resides in |
| 7 | Head of the family | Person of authority within the family who makes important decisions |
| 8 | Mother’s education | Level of education that the participant’s mother has completed |
| 9 | Family income | Total amount of money that the family earns in a month |
| 10 | Marital status | Participant’s marital status at the time of interview |
| 11 | Age at marriage | Participant’s age at first marriage |
| 12 | Sexual relationship status | Participant’s status based on whether she has ever had a sexual relationship or not |
| 13 | Age at first sexual relationship | Participant’s age at first sexual relationship |
| 14 | Use of contraceptives | Participant’s status based on whether she has ever used contraceptives or not |
| 15 | Method of diagnosis of pregnancy | The method used to diagnose the pregnancy (symptomatic or health checkup) |
| 16 | Number of ANC Visits | The number of antenatal visits the patient had during her pregnancy |
| 17 | Iron consumption | Participant’s status based on whether she took iron supplement during her pregnancy or not |
| 18 | Calcium consumption | Participant’s status based on whether she took calcium supplement during her pregnancy or not |
| 19 | Outcome of previous pregnancy | Outcome of previous pregnancy based on whether the participant had a live birth or not |
| 20 | Neonatal health issues in previous pregnancy | Status of the neonate during previous pregnancy based on whether it had any health problems after birth or not |
| 21 | Maternal health issues in previous pregnancy | Status of the mother during previous pregnancy based on whether she had any health problems after delivery or not |
