# Supplementary material for: Factors associated with adolescent pregnancy among Chepang women and their health-seeking behavior in Ichchhakamana rural municipality of Chitwan district
Source: PLoS One. 2024 Mar 28;19(3):e0301261. doi: 10.1371/journal.pone.0301261 (PMC10977708; doi:10.1371/journal.pone.0301261)
Supplement: S2 Table — (DOCX) [file pone.0301261.s002.docx]

**S2 Table. Collinearity Statistics.**

| **Independent Variables** | **Collinearity Statistics** | |
| --- | --- | --- |
|  | **Tolerance** | **VIF** |
| Religion | .988 | 1.012 |
| Participant’s Education Level | .809 | 1.237 |
| Family Type | .802 | 1.247 |
| Head of Family | .699 | 1.431 |
| Monthly Family Income | .642 | 1.559 |
| Mother's Education Level | .658 | 1.520 |
| **Dependent Variable** | Current pregnancy status | |
