# Supplementary material for: Factors associated with adolescent pregnancy among Chepang women and their health-seeking behavior in Ichchhakamana rural municipality of Chitwan district
Source: PLoS One. 2024 Mar 28;19(3):e0301261. doi: 10.1371/journal.pone.0301261 (PMC10977708; doi:10.1371/journal.pone.0301261)
Supplement: Attachment — Submitted filename: PONE-D-23-26050.docx [file pone.0301261.s008.docx]

PLOS ONE

**Factors Associated with Adolescent Pregnancy among Chepang Women and Their Health-Seeking Behavior in Ichchhakamana Rural Municipality of Chitwan District**

--Manuscript Draft--

| **Manuscript Number:** | PONE-D-23-26050 |
| --- | --- |
| **Article Type:** | Research Article |
| **Full Title:** | Factors Associated with Adolescent Pregnancy among Chepang Women and Their Health-Seeking Behavior in Ichchhakamana Rural Municipality of Chitwan District |
| **Short Title:** | Adolescent Pregnancy and Health-seeking Behavior among Chepang Women |
| **Corresponding Author:** | Smriti Pant, MD in Community Medicine Tribhuvan University Institute of Medicine Kathmandu, Nepal NEPAL |
| **Keywords:** | Adolescent Pregnancy; Chepang; Health Seeking. |
| **Abstract:** | Adolescent pregnancy is a critical public health issue, particularly in developing regions like Nepal, where it poses significant risks to maternal and child health and perpetuates the cycle of poverty. This study focused on the marginalized Chepang community, which is endangered and faces unique challenges. The study aimed to assess the factors associated with adolescent pregnancy among Chepang women in Ichchhakamana Rural Municipality, Chitwan, Nepal, and also explored their reproductive health-seeking behavior.  A cross-sectional analytical study was conducted with 217 Chepang women aged 15- 20 years, and data was collected through face-to-face interviews using a structured questionnaire. The prevalence of current adolescent pregnancy was 8.3%, while one- fourth had experienced prior pregnancies during their adolescence. Factors significantly associated with adolescent pregnancy included lack of education among the women and their mothers, as well as living in joint families.  Addressing adolescent pregnancy requires comprehensive strategies that prioritize education and raise awareness about reproductive health among marginalized communities like the Chepang. |
| **Order of Authors:** | Smriti Pant, MD in Community Medicine |
|  | Saugat Koirala, MS Gynecology and Obstetrics |
|  | Anand Prasad Acharya, MBBS |
|  | Pranil Man Singh Pradhan, MD in Community Medicine and Tropical Diseases |
| **Additional Information:** |  |
| **Question** | **Response** |
| **Financial Disclosure**  Enter a financial disclosure statement that describes the sources of funding for the work included in this submission. Review the [submission guidelines](http://journals.plos.org/plosone/s/submission-guidelines#loc-financial-disclosure-statement) for detailed requirements. View published research articles from [*PLOS ONE*](http://journals.plos.org/plosone/) for specific examples.  This statement is required for submission and **will appear in the published article** if the submission is accepted. Please make sure it is accurate. | The author(s) received no specific funding for this work. |

| **Unfunded studies**  Enter: *The author(s) received no specific funding for this work.*  **Funded studies**  Enter a statement with the following details:   - Initials of the authors who received each award - Grant numbers awarded to each author - The full name of each funder - URL of each funder website - Did the sponsors or funders play any role in the study design, data collection and analysis, decision to publish, or preparation of the manuscript? - **NO** - Include this sentence at the end of your statement: *The funders had no role in study design, data collection and analysis, decision to publish, or preparation of the manuscript.* - **YES** - Specify the role(s) played. |  |
| --- | --- |
| * typeset |  |
| **Competing Interests**  Use the instructions below to enter a competing interest statement for this submission. On behalf of all authors, disclose any [competing interests](http://journals.plos.org/plosone/s/competing-interests) that could be perceived to bias this work—acknowledging all financial support and any other relevant financial or non- financial competing interests.  This statement is required for submission and **will appear in the published article** if the submission is accepted. Please make sure it is accurate and that any funding sources listed in your Funding Information later in the submission form are also declared in your Financial Disclosure statement.  View published research articles from  [*PLOS ONE*](http://journals.plos.org/plosone/) for specific examples. | The authors have declared that no competing interests exist. |

| **NO authors have competing interests**  Enter: *The authors have declared that no competing interests exist.*  **Authors with competing interests**  Enter competing interest details beginning with this statement:  *I have read the journal's policy and the authors of this manuscript have the following competing interests: [insert competing interests here]* |  |
| --- | --- |
| * typeset |  |
| **Ethics Statement**  Enter an ethics statement for this submission. This statement is required if the study involved:   - Human participants - Human specimens or tissue - Vertebrate animals or cephalopods - Vertebrate embryos or tissues - Field research   Write "N/A" if the submission does not require an ethics statement.  General guidance is provided below. Consult the [submission guidelines](http://journals.plos.org/plosone/s/submission-guidelines#loc-guidelines-for-specific-study-types) for detailed instructions. **Make sure that all information entered here is included in the Methods section of the manuscript.** | -Approval has been obtained from Nepal Health Research Council ,Nepal  -ERB Registration Number is 329/2021 P  -Informed consent from the participants was obtained in written form |

| **Format for specific study types**  **Human Subject Research (involving human participants and/or tissue)**   - Give the name of the institutional review board or ethics committee that approved the study - Include the approval number and/or a statement indicating approval of this research - Indicate the form of consent obtained (written/oral) or the reason that consent was not obtained (e.g. the data were analyzed anonymously)   **Animal Research (involving vertebrate animals, embryos or tissues)**   - Provide the name of the Institutional Animal Care and Use Committee (IACUC) or other relevant ethics board that reviewed the study protocol, and indicate whether they approved this research or granted a formal waiver of ethical approval - Include an approval number if one was obtained - If the study involved *non-human primates*, add *additional details* about animal welfare and steps taken to ameliorate suffering - If anesthesia, euthanasia, or any kind of animal sacrifice is part of the study, include briefly which substances and/or methods were applied   **Field Research**  Include the following details if this study involves the collection of plant, animal, or other materials from a natural setting:   - Field permit number - Name of the institution or relevant body that granted permission |  |
| --- | --- |
|  |  |
| **Data Availability**  Authors are required to make all data underlying the findings described fully available, without restriction, and from the time of publication. PLOS allows rare exceptions to address legal and ethical concerns. See the [PLOS Data Policy](http://journals.plos.org/plosone/s/data-availability) and [FAQ](http://journals.plos.org/plosone/s/data-availability#loc-faqs-for-data-policy) for detailed information. | Yes - all data are fully available without restriction |

| A Data Availability Statement describing where the data can be found is required at submission. Your answers to this question constitute the Data Availability Statement and **will be published in the article**, if accepted. |  |
| --- | --- |
| **Important:** Stating ‘data available on request from the author’ is not sufficient. If your data are only available upon request, select ‘No’ for the first question and explain your exceptional situation in the text box. |  |
| Do the authors confirm that all data underlying the findings described in their manuscript are fully available without restriction? |  |
| **Describe where the data may be found in full sentences. If you are copying our sample text, replace any instances of XXX with the appropriate details.**   - If the data are **held or will be held in a public repository**, include URLs, accession numbers or DOIs. If this information will only be available after acceptance, indicate this by ticking the box below. For example: *All XXX files are available from the XXX database (accession number(s) XXX, XXX.*). - If the data are all contained **within the manuscript and/or Supporting Information files**, enter the following: *All relevant data are within the manuscript and its Supporting Information files.* - If neither of these applies but you are able to provide **details of access elsewhere**, with or without limitations, please do so. For example:   *Data cannot be shared publicly because of [XXX]. Data are available from the XXX Institutional Data Access / Ethics Committee (contact via XXX) for researchers who meet the criteria for access to confidential data.*  *The data underlying the results presented in the study are available from (include the name of the third party* | All relevant data are within the manuscript and its Supporting Information files. |

| *and contact information or URL).*   - This text is appropriate if the data are owned by a third party and authors do not have permission to share the data.   * typeset |  |
| --- | --- |
| Additional data availability information: |  |

Manuscript	[Click here to access/download;Manuscript;Manuscript_Plos One Chepang 15th Aug.docx](https://www.editorialmanager.com/pone/download.aspx?id=33945679&guid=2fde431f-cb81-4217-ae5d-d33f9346fe92&scheme=1)

# 1 Title:

2 Factors Associated With Adolescent Pregnancy among Chepang Women and Their Health-
3 Seeking Behavior in Ichchhakamana Rural Municipality Of Chitwan District
4 Short title: Adolescent Pregnancy and Health-seeking Behavior among Chepang Women

5

# 6 Authors:

7 **Smriti Pant**
8 Assistant Professor, Department of Community Medicine, Maharajgunj Medical Campus,
9 Maharjgunj, Kathmandu, Nepal
10 Email: [drsmritipant@gmail.com](mailto:drsmritipant@gmail.com)

# 11 Saugat Koirala

12 Lecturer, Department of Gynecology and Obstetrics, KIST Medical College, Imadol, Lalitpur,
13 Nepal

# 14 Anand Prasad Acharya

15 Senior House officer, Department of Medicine, Frimley park Hospital, Frimley, Camberley, UK

# 16 Pranil Man Singh Pradhan

17 Associate Professor, Department of Community Medicine, Maharajgunj Medical Campus,
18 Maharjgunj,Kathmandu, Nepal

19

20

21

22

23

# 24 Abstract:

25 Adolescent pregnancy is a critical public health issue, particularly in developing regions like
26 Nepal, where it poses significant risks to maternal and child health and perpetuates the cycle of
27 poverty. This study focused on the marginalized Chepang community, which is endangered and
28 faces unique challenges. The study aimed to assess the factors associated with adolescent
29 pregnancy among Chepang women in Ichchhakamana Rural Municipality, Chitwan, Nepal, and
30 also explored their reproductive health-seeking behavior.
31 A cross-sectional analytical study was conducted with 217 Chepang women aged 15-20 years,
32 and data was collected through face-to-face interviews using a structured questionnaire. The
33 prevalence of current adolescent pregnancy was 8.3%, while one-fourth had experienced prior
34 pregnancies during their adolescence. Factors significantly associated with adolescent pregnancy
35 included lack of education among the women and their mothers, as well as living in joint
36 families.
37 Addressing adolescent pregnancy requires comprehensive strategies that prioritize education and
38 raise awareness about reproductive health among marginalized communities like the Chepang.

39

40

41

42

43

44

# 45 Introduction:

46 Adolescence is a transitional stage of physical and psychological development in the age group of
47 10 and 19 years.^1^ Pregnancy during adolescence is a major contributor of maternal and child
48 mortality, and also leads to a vicious cycle of ill health and poverty. ^2^ Adolescent pregnancy occurs
49 in estimated 21 million in developing regions and approximately 12 million of them give birth.^2^
50 Babies born to these adolescent mothers account for nearly 11% of births worldwide with 95%
51 occurring in developing countries like Nepal. ^3^ Two million of these births are from girls under 15
52 years of age.^4^ Pregnancy and childbirth complications are the leading cause of death globally
53 among girls aged 15–19 years , with low- and middle-income countries accounting for 99% of
54 global maternal deaths of women aged 15–49 years.^5^ Adolescent mothers aged 10–19 years face
55 higher risks of eclampsia, puerperal endometritis and systemic infections than women aged 20–24
56 years.^6^ Other consequences are unsafe abortion, still birth and new born deaths. ^2^ There are many
57 factors and determinants associated with adolescent pregnancy, explained in many literatures.^3,^ ^7,^ ^8^
58 In case of marginalized community like Chepang community, factors associated with adolescent
59 pregnancy are household wealth index, ethnicity, religion, sex of the household head, maternal
60 education, literacy level and occupation.^8^ Nepal Demographic and Health Survey (NDHS) 2016
61 showed that the country’s adolescent birth rate was 88 per 1000 women, with 17% of girls aged
62 15–19 years already mothers of at least one child.^9^ This survey also shows that factors like poor
63 household wealth index, being Dalit or Madhesi , and unemployment among women was
64 associated with higher odds of adolescent pregnancies. In contrast, adolescent pregnancy was
65 significantly lower among educated women, and women with access to information on public
66 health issues. ^8^ But there is no study available to compare this data with Chepang women's rate of
67 adolescent pregnancy.
68 Chepang community is a marginalized and endangered community of Nepal. This community has
69 around 69000 inhabitants in Nepal out of which 8357 population live in Ichchhakamana Rural
70 Municipality of Chitwan district.^10^Around 1900 of them are females of reproductive age group
71 (15-49 years). ^10^ An article about adolescent pregnancy in Chepang women in 2016 showed that
72 about 58.1 percent of the respondents have their first child birth in their adolescent age.^11^It also
73 showed that complications of adolescent pregnancy among them were preterm labour, prolonged
74 labour, miscarriage and postpartum haemorrhage.^11^
75 Nepal Demographic Health Survey (NDHS 2016) estimates show that median age of marriage for
76 women if 17.9 years and the median age of first sexual intercourse is also 17.9 years.^9^ These are
77 risk factors for adolescent pregnancy. A study in Korak VDC in Chitwan showed that the average
78 age of marriage in Chepang females is 15.48 years and mean age of first child birth was 16.95
79 years.^11^ Similarly, another study in Dhading district of Nepal illustrated that the mean age of
80 marriage and first pregnancy was 16.7 years and 17.7 years respectively.^12^ Additionally, they also
81 have low contraceptive prevalence rate (49.9%) among married Chepang women. ^12^ Studies have
82 shown that there are many strategies adopted by WHO and Nepal to reduce adolescent pregnancy.
83 A global strategy for women's and children's health launched by United Nations Secretary General
84 in 2010, stresses the importance of addressing the health and welfare of adolescent girls in order
85 to achieve the fifth millennium development goal on maternal mortality reduction.^3^ WHO has
86 published guidelines on preventing early pregnancy and poor reproductive outcomes among
87 adolescent women in the developing countries like Nepal.^3^ Nepal has developed national
88 reproductive health strategy in 1998, in which adolescent reproductive health was identified as a
89 critical component of the integral health package and thus an important indicator of the nations
90 over all health status.^13^ This study aimed to assess the factors associated with adolescence
91 pregnancy among Chepang women in of Ichchhakamana Rural Municipality, Chitwan. It also
92 assessed their reproductive health seeking behavior.

# 93 Materials and methods:

## 94 Study design and population

95 A cross-sectional analytical study was conducted in three wards of Ichchhakamana Rural
96 Municipality, Chitwan. The data collection was done between July 2021 to October 2021. The
97 study participants were Chepang women aged 15-20 years living at the study site. All women who
98 gave consent to participate in the study were included while those who were unable to answer due
99 to language barrier were excluded from the study. The sample size calculation was done using the
100 formula, sample size=z^2^. p. q/ e^2^. Considering the value of p as 0.17 ^9^ sample size was calculated

101	as 217.

102 The study started with purposive selection of Ichchhakamana Rural Municipality as the study site.
103 The municipality consists of seven wards. At the first stage, three wards were chosen randomly
104 for the study using lottery method. Next, the study site was visited and the proposed participants
105 in the Chepang community were informed about the objectives of the research and its expected
106 outcome. After getting approval for engagement, the required sample size was taken equally from
107 all wards using sampling intervals (72 from ward 1 and 3 and 73 from ward 6 ). First house from
108 respective ward was selected randomly and rest of the houses were selected consecutively until
109 required sample size was reached. If more than one, participant meeting the eligibility criteria were
110 found at selected households then all of them were interviewed.

## 111 Study variables and data collection

112 The dependent variable was adolescent pregnancy while independent variables included socio-
113 demographic, sexual and reproductive characteristics of the participants. Other variables included
114 health seeking behavior like number of antenatal visits, age at first intercourse etc.
115 Adolescent Pregnancy was defined as any women aged 15-20 years who have had a live birth
116 within the last 1 year or women aged 15-19 years who are pregnant with their first child during the
117 time of the study.
118 The data was collected with the help of a questionnaire by using face to face interview technique.
119 The questionnaire consisted information regarding socio-demographic details, sexual and
120 reproductive characteristics, health seeking behaviour etc. Extensive literature review was done
121 for making the questionnaire. The questionnaire was shared with content experts for content
122 validity. Face validity of the questionnaire was maintained by discussion with colleagues.
123 Pretesting was done in 10 % of sample size (among the population in the selected ward who do
124 not belong to Chepang ethnicity) and necessary modifications were made to the questions. As the
125 questionnaire demands recalling information for the past 1 year, there is a chance of recall bias.

## 126 Statistical analysis

127 The collected data was entered and analyzed using IBM SPSS version 20. First of all, the
128 prevalence of adolescent pregnancy was calculated. Descriptive statistical tools like frequency,
129 and percentage were used to express the results. Pearson chi-square test, Fischer exact test were
130 used for bivariate analysis to determine the presence of association between the dependent and
131 independent variables. Crude odds ratio (COR) at 95% confidence interval (95% CI) was
132 calculated to see the magnitude of association with independent variables. In multivariate analysis,
133 binary logistic regression was carried out. Independent variables associated with current pregnancy
134 status with a significance level of less than 0.20 (p-value< 0.20) were adjusted among themselves
135 to calculate corresponding adjusted odds ratio (AOR). All tests were done with the significance
136 level set at 5% (p-value <0.05).In the assessment of multi-collinearity, the Variance Inflation
137 Factor (VIF) was determined to be less than 10. This confirms the absence of any interconnection
138 among the independent variables.

## 139 Ethical Consideration

140 Ethical approval was taken from Nepal Health Research Council (NHRC). The office of
141 Ichchhakamana rural municipality was contacted and a letter of support for community
142 engagement was obtained. After this, the ward chairpersons of the selected wards (wards 1, 3 and
143 6) were contacted and permission was taken to conduct the research. Next, a written consent was
144 taken from the respondents or their parents. If the respondents were married at the time of
145 interview, written consent was taken from the respondents themselves. However, if the respondent
146 was unmarried, written consent was taken from the parent/guardian. The respondents’ participation
147 in the study was voluntary and their confidentiality was maintained. The information is kept safely
148 in the computer with proper password to disallow any fallacy in conduction of the study.

# 149 Results

150 There were total 217 participants. The features of the participants are as follows:

# 151 Socio-demographic characteristics of the participants

152 They were aged between 15 to 20 years of age. Their mean age was 17.6 years with standard
153 deviation of 1.7 years. Most of the participants were aged 18 years (19.8%) followed by 20 years
154 of age. (Table 1) Furthermore, most of the participants were Hindu (85.3%) and majority of the
155 women had studied till Primary level (67.7%). (Table 1) Similarly, 30 % were involved in farming.
156 (Table 1)

# 157 Table 1: Socio-demographic characteristics of the participants

| **Variables** | **Frequency** | **Percentage** |
| --- | --- | --- |
| **Socio-demographic characteristics** |  |  |
| **Age (in years)** |  |  |
| 15 | 35 | 16.1 |
| 16 | 41 | 18.9 |
| 17 | 22 | 10.1 |
| 18 | 43 | 19.8 |
| 19 | 35 | 16.0 |
| 20 | 41 | 18.9 |
| Total | 217 | 100.0 |
| **Religion** |  |  |
| Hindu | 185 | 85.3 |
| Christian | 32 | 14.7 |
| Total | 217 | 100.0 |
| **Education** |  |  |
| Never been to school | 59 | 27.2 |
| Primary Level | 147 | 67.7 |
| SEE pass | 7 | 3.2 |
| Intermediate level | 4 | 1.8 |
| Total | 217 | 100.0 |
| **Occupation** |  |  |
| Housewife | 45 | 20.7 |
| Farmer | 70 | 32.3 |
| Government Job | 1 | 0.5 |
| Private Job | 3 | 1.4 |
| Laborer | 54 | 24.9 |
| Student | 44 | 20.3 |
| Total | 217 | 100.0 |
| **Family Characteristics** |  |  |
| **Family Type** |  |  |
| Nuclear | 119 | 54.8 |
| Joint Family | 98 | 45.2 |
| Total | 217 | 100.0 |
| **Head of the Family** |  |  |
| Male | 159 | 73.3 |
| Female | 58 | 26.7 |
| Total | 217 | 100.0 |
| **Mother’s educational status** |  |  |
| Never been to school | 111 | 51.2 |
| Primary Level | 106 | 48.8 |
| Total | 217 | 100.0 |
| **Family Income** |  |  |
| Less than Rs. 15000 | 78 | 35.9 |
| More than or equal to Rs. 15000 | 139 | 64.1 |
| Total | 217 | 100.0 |
| **Sexual and Reproductive health characteristics**  **Marital status** | | |
| Single | 113 | 52.0 |
| Married | 103 | 47.5 |
| Widower | 1 | 0.5 |
| Total | 217 | 100.0 |
|  |  |  |

| **Age at Marriage (in years)** | | | |
| --- | --- | --- | --- |
| 14 | | 4 | 3.8 |
| 15 | | 25 | 24.0 |
| 16 | | 27 | 26.0 |
| 17 | | 10 | 9.6 |
| 18 | | 29 | 28.0 |
| 19 | | 4 | 3.8 |
| 20 | | 5 | 4.8 |
| Total | | 104 | 100.0 |
| **Sexual relationship status**  Had sexual relations | | 104 | 47.9 |
| Never had sexual relations | | 113 | 52.1 |
| Total | | 217 | 100.0 |
| **Age at first sexual relationship(in years)**  Less than15 | | 25 | 24.0 |
| 15 to 18 | | 67 | 64.4 |
| More than 18 | | 12 | 11.6 |
| Total | | 104 | 100.0 |
| **Use of contraceptive**  Ever used contraceptive | | 15 | 14.4 |
| Never used contraceptive | | 89 | 85.6 |
| Total | | 104 | 100.0 |
| **Reasons for not using contraceptives** | | | |
|  | Not feasible | 38 | 42.7 |
|  | Desire to get pregnant | 32 | 36.0 |
|  | Lack of awareness | 14 | 15.7 |
|  | Living separately from spouse | 3 | 3.4 |
|  | Family influence | 2 | 2.2 |
|  | Total | 89 | 100.0 |
| 158 |  |  |  |

159 **Characteristics of family*:***
160 More than half (54.8%) of the families were nuclear. (Table 1) In about three quarters (73.3%) of
161 the families, the head of the household was a male. Additionally, 64 % of the families had an
162 income of more than NRs. 15,000. (Table 1) Similarly, almost half of the participant’s mother had
163 never been to school while the rest had studied till Primary level. (Table 1)

# 164 Reproductive and sexual health characteristics of the participants:

## 165 Sexual health characteristics:

166 Almost half (47.5%) of the participants were married and majority (28%) of them were married at
167 18 years of age. (Table 1) The mean age of marriage was 16.84 years with standard deviation of
168 1.52 years. About 48% of the participants stated that they have been sexually active. (Table 1) For
169 majority of them (64.4%), the age of first sexual contact was 15 to 18 years and only about 14%
170 had used contraceptive devices. (Table 1) The most common reason behind not using contraceptive
171 methods were its use not being feasible (42.7%), desire to get pregnant (36%) and lack of
172 awareness (15.7%) about the methods.

## 173 Features of Current Adolescent Pregnancy:

174 Among the participants, 8.3% were currently pregnant. Almost all except one pregnancy was
175 unplanned. But all of the pregnant women were happy about their pregnancy. While 72% of the
176 pregnant women considered amenorrhea as an indicator of pregnancy, the rest relied on health
177 checkup. Amongst the pregnant, most (44.5%) were in their second trimester. (Table 2)
178 Furthermore, 50% of them were taking iron tablets, but only 22% were taking calcium tablets.
179 (Table 2) Regarding the tests done during pregnancy, 83.3 % of the women had physical
180 examination,66.7% had a urine examination, 22% had an ultrasonography, 11 % had done blood
181 tests , but only 11 % of them had done all of these.

# 182 Table 2: Features of current pregnancy

| **Variables** | **Frequency** | **Percentage** |
| --- | --- | --- |
| **Period of gestation** |  |  |
| Up to 13 weeks | 6 | 33.3 |
| 13 to 28 weeks | 8 | 44.5 |
| More than 28 weeks | 4 | 22.2 |
| Total | 18 | 100.0 |
| **Diagnosis of pregnancy** | | |
| Amenorrhea | 13 | 72.0 |
| Health check up | 5 | 28.0 |
| Total | 18 | 100.0 |
| **Visits to health facilities after pregnancy** | | |
| Once | 9 | 50.0 |
| Twice | 3 | 16.7 |
| Thrice | 4 | 22.2 |
| Never | 2 | 11.1 |
| Total | 18 | 100.0 |
| **Taking iron** | | |

Yes	9	50.0

| No | | 9 | 50.0 |
| --- | --- | --- | --- |
| Total | | 18 | 100.0 |
| **Taking calcium** | | | |
|  | Yes | 4 | 22.2 |
|  | No | 14 | 77.8 |
|  | Total | 18 | 100.0 |
| 183 |  |  |  |

## 184 Features of previous adolescent pregnancy:

185 Almost a quarter (24%) of the participants had a previous pregnancy. Among them, 79 % had live
186 births and majority of them did not have any health issues among mothers (90.2%) or the neonates
187 (82.7%) at the end of the pregnancy. (Table 3)

# 188 Table 3: Features of previous adolescent pregnancy

**Variables	Frequency	Percentage**

**Outcome of pregnancy**

| Live birth | | 41 | 79.0 |
| --- | --- | --- | --- |
| Miscarriage/still birth /IUFD | | 11 | 21.0 |
| Total | | 52 | 100.0 |
| **Health issue in neonates**  Yes | | 4 | 9.8 |
| No | | 37 | 90.2 |
| Total | | 41 | 100.0 |
| **Health issue in mothers after birth** | | | |
|  | Yes | 9 | 17.3 |
|  | No | 43 | 82.7 |
|  | Total | 52 | 100.0 |
| 189 |  |  |  |

# 190 Association of adolescent pregnancy with socio-demographic and family variables:

191 Higher percentage of participants who were pregnant belonged to Hindu religion. But this
192 relationship was not statistically significant. (Table 4). The study showed that those adolescents
193 who had never been to school had 2.9 times more chance of being pregnant compared to those
194 who had been to at least primary school (COR (95%CI): 2.9[1.1-7.9]) and this relationship was
195 statistically significant.(p <0.05) (Table 4). Similarly, those who lived in joint families had a higher
196 chance of being pregnant compared to nuclear families (COR (95%CI): 4.8 [1.5-15.0]) and this
197 relationship was also statistically significant (p<0.05) (Table 4). Among the pregnant adolescents
198 most had females as head of the family and lived in families who earned less than NRs. 15,000.
199 But these associations were not statistically significant. Furthermore, adolescents with mothers
200 who never went to school had 3.6 times more risk of being pregnant compared to those who had
201 primary level education (COR, 95% CI: 3.68[1.17-11.57]) and this association was statistically
202 significant (p<0.5).
203 After adjustment for confounders only living in joint family (AOR, 95% CI: 3.4[1.01-11.74]) was
204 significantly associated with higher occurrence of adolescent pregnancy.(p<0.05)

# 205 Table 4: Association of adolescent pregnancy with socio-demographic and family variables:

| **Variables Pregnant** | | **Not Pregnant** | **Total** | **COR[95 %CI]** | **p value** | **AOR[95 %CI]** | **p value** |
| --- | --- | --- | --- | --- | --- | --- | --- |
| **Religion** | | | | | | | |
| Hindu | 16(8.6) | 169(91.4) | 185(100.0) | 1.4 [0.3-6.4] | 1.0^a^ | - - | |
| Christian | 2(6.3) | 30(93.8) | 32(100.0) | Ref |  | - - | |
| **Participant’s Education level** | | | | | | | |
| Never been to school | 9(15.3) | 50(84.7) | 59(100.0) | 2.9 [1.1-7.9] | 0.048^a*^ | 1.8[0.61-5.5] | 0.28 |
| Primary level and above | 9(5.7) | 149(94.3) | 158(100.0) | Ref |  |  |  |
| **Family Type** |  |  |  |  |  |  |  |
| Joint | 14(14.3) | 84(85.7) | 98 (100.0) | 4.8 [1.5-15.0] | 0.004^b*^ | 3.4[1.01-11.74] | 0.049* |
| Nuclear | 4(3.4) | 115(96.6) | 119(100.0) | Ref |  | Ref |  |
| **Head of the Family** |  |  |  |  |  |  |  |
| Female | 8(13.8) | 50(86.2) | 58(100.0) | 2.38[0.89-6.37] | 0.095^a^ | 0.91[0.28-2.8] | 0.86 |
| Male | 10(6.3) | 149 (93.7) | 159(100.0) | Ref |  | Ref |  |
| **Monthly Family Income** |  |  |  |  |  |  |  |
| < NRs. 15,000 | 8(10.3) | 70(89.7) | 78 (100.0) | 1.47[0.56-3.91] | 0.43^b^ | **-** | **-** |
| > NRs. 15,000 | 10(7.2) | 129(92.8) | 139 (100.0) | Ref |  | - | - |
| **Mother’s Education level** |  |  |  |  |  |  |  |
| Never been to school | 14(12.6) | 97(84.4) | 111(100.0) | 3.68[1.17-11.6] | 0.018^b*^ | 2.23[0.64-8.18] | 0.2 |
| Primary level and above | 4(3.8) | 102(96.2) | 106(100.0) | Ref |  | Ref |  |
| 206  207 *^a^ Fischer exact test* |  |  |  |  |  |  |  |

208 *^b^ Chi-square test*
209 **statistically significant at p<0.05*
210 210
211 211

# 212 Discussion:

213 This study aimed to assess the factors associated with adolescence pregnancy among Chepang
214 women in of Ichchhakamana Rural Municipality, Chitwan. It also assessed their reproductive
215 health seeking behavior.
216 Among the 217 participants, majority of the women had studied till Primary level (67.7%) while
217 the rest had no education. This is in contrast to a study among 80 Chepang women in Dhading
218 district of Nepal, where almost 26% percent had studied till primary level, but almost 30 percent
219 had education of secondary level or higher.^12^ Similarly another study in Korak VDC, Nepal among
220 148 Chepang women showed that 36.4% of the women had primary level education and almost 5
221 % had secondary level education.^11^ Furthermore, the most common occupation among the
222 participants in our study was farming, which was similar to the study in Korak VDC but in contrast
223 to the finding in Dhading where most of the participants were housewives.^12^ In our study more
224 than half of the families were nuclear. This finding was different than other studies conducted in
225 Nepal. ^11,^ ^12^ Additionally, in this study, most of the families had an income of more than NRs.
226 15,000. This was in contrast to another study in Nepal.^12^
227 In our study, the mean age of marriage was 16.84 years. This finding is similar to other studies
228 conducted in Nepal. ^11,^ ^12^ In our study almost half of the women stated that they were sexually
229 active. For majority (64.4%) of them, the age of first sexual contact was 15 to 18 years and only
230 about 14% had used contraceptive devices. The most common reason behind not using
231 contraceptive methods were its use not being feasible (42.7%), desire to get pregnant (36%) and
232 lack of awareness (15.7%) about the methods.
233 In our study, the prevalence of adolescents who were currently pregnant was 8.3%. This is in
234 contrast to the results of a Systematic Review and Meta-analysis conducted in Nepal which showed
235 the prevalence to be a bit higher (13.2%). ^14^ Similarly, a report by UNICEF showed that, in South
236 Asia region adolescent marriage and pregnancy was highest among Nepalese and Bangladeshi
237 girls.^15^ Nevertheless, it is noteworthy to highlight that approximately one-fourth of the participants
238 disclosed a history of prior pregnancies. Given the age range of our participants, spanning from 15
239 to 20 years, this observation suggests that these women experienced teenage pregnancies at some
240 stage during their adolescence.
241 Our study showed that those adolescents who had never been to school had 2.9 times more chance
242 of being pregnant compared to those who had been to at least primary school (COR (95%CI):
243 2.9[1.1-7.9]). This finding is supported by World Bank estimates which states that more education
244 for girls can result in delayed pregnancy. ^16^ Similarly, several other researches have illustrated that
245 marrying at a young age and early pregnancy were both results of low level of education among
246 the adolescent girls. ^15,17,^ ^18^ Education empowers women by providing them with knowledge about
247 reproductive health and family planning, leading to more informed choices. ^19^ This may result in
248 delayed marriages, encourage aspirations beyond early motherhood, and enhance access to
249 resources and healthcare.
250 Furthermore, in our study those who lived in joint families had a higher chance of being pregnant
251 compared to nuclear families (COR (95%CI): 4.8 [1.5-15.0]) and this relationship was also
252 statistically significant (p<0.05) (Table 7). The higher likelihood of pregnancy in joint families
253 can be attributed to different reasons. Residing in joint families can result in limited personal
254 freedom and the perpetuation of traditional beliefs, ^20^ often leading to early age at marriage and
255 childbirth. However, it also offers the advantage of a larger support system and sharing of
256 responsibilities, ^21^ which may promote pregnancies by creating a more conducive environment for
257 giving birth to and raising children.
258 Among the pregnant adolescents most had females as head of the family and lived in families who
259 earned less than NRs. 15,000. But these associations were not statistically significant.
260 Furthermore, adolescents with mothers who never went to school had 3.6 times more risk of being pregnant
261 compared to those who had at least primary level education (COR, 95% CI: 3.68[1.17-11.57]) and this
262 association was statistically significant (p<0.5). Mothers’ education has been associated with reduced level
263 of fertility and increased age at first birth for her offspring.^22^ Hence, this heightened risk of pregnancy
264 among the adolescents may be attributed to limited knowledge of reproductive health among the
265 uneducated mothers.
266 Our study had few limitations. Since participants had to recollect memories in order to respond to
267 the questions, a potential recall bias is there. Similarly, due to the design of our study we were
268 unable to assess the temporality of association.

# 269 Conclusion:

270 Teenage pregnancy was high among the Chepang women in Ichchhakamana Rural Municipality.
271 It was associated with low level of education among women and their mothers and their family
272 type. Addressing adolescent pregnancy requires comprehensive strategies prioritizing education
273 and reproductive health awareness.

# 274 Acknowledgments:

275 We wish to extend our profound appreciation to all adolescents and their families for their
276 participation and invaluable support. Additionally, we would like to recognize the assistance
277 rendered by the ward representatives of wards one, three, and six within the Ichchhakamana Rural
278 Municipality. Their authorization for data collection and cooperation are highly acknowledged and
279 appreciated.

# 280 Author Contributions:

281 **Conceptualization:** Smriti Pant, Saugat Koirala, Anand Prasad Acharya, Pranil Man Singh
282 Pradhan.
283 **Investigation:** Smriti Pant, Saugat Koirala, Anand Prasad Acharya
284 **Data curation:** Smriti Pant
285 **Formal analysis:** Smriti Pant
286 **Methodology:** Smriti Pant, Saugat Koirala, Anand Prasad Acharya, Pranil Man Singh Pradhan.
287 **Resources**: Smriti Pant
288 **Supervision:** Smriti Pant, Pranil Man Singh Pradhan
289 **Validation:** Smriti Pant, Pranil Man Singh Pradhan.
290 **Writing – original draft:** Smriti Pant
291 **Writing – review & editing**: Smriti Pant, Saugat Koirala Pranil Man Singh Pradhan.
292 292

# 293 References:

294 1. World Health Organization. Adolescent health in the South-East Asia Region [Internet].
295 Geneva:	World	Health	Organization.	2015	[cited	2022	Dec	12].	Available	from:
296 <https://www.who.int/southeastasia/health-topics/adolescent-health>
297 2. Darroch JE, Woog V, Bankole, Ashford. Adding it up: costs and benefits of meeting the
298 contraceptive needs of adolescents. New York: Guttmacher Institute; 2016. Available from:
299 <https://www.guttmacher.org/sites/default/files/report_pdf/adding-it-up-adolescents-report.pdf>
300 3 .World Health Organization*. Preventing early pregnancy and poor reproductive outcomes*
301 *among adolescents in developing countries [Internet].* Geneva: World Health Organization.
302 2011[cited	2022	Dec	12].Available	from:
303 <https://www.who.int/publications/i/item/9789241502214>
304 4. Williamson N. Motherhood in Childhood:Facing the challenge of adolescent pregnancy
305 [Internet].Newyork: United Nations Fund for Population.2016[cited 2022 Dec 14]. Avaiilable
306 from: <https://www.unfpa.org/sites/default/files/pub-pdf/EN-SWOP2013-final.pdf>
307 5.	Neal S, Matthews Z, Frost M, Fogstad H, Camacho A, Laski L. Childbearing in adolescents
308 aged 12–15 years in low resource countries: a neglected issue. New estimates from demographic
309 and household surveys in 42 countries. Acta obstetricia et gynecologica Scandinavica.

310	2012;91(9):1114-8. [PubMed](https://pubmed.ncbi.nlm.nih.gov/22620274/) [DOI](https://doi.org/10.1111/j.1600-0412.2012.01467.x)

311 6.	World Health Organization. Adolescent Pregnancy[Internet]. Geneva: World Health
312 Organization; 2020 [cited 2022 Jan 2023].Available from: <https://shorturl.at/dpsAZ>
313 7.	Yohannes A, Anteneh Y, Telake A. Prevalence and factors associated with teenage
314 pregnancy, Northeast Ethiopia, 2017: a cross-sectional study. Journal of pregnancy. 2018; Article
315 ID 1714527. [Google Scholar](https://scholar.google.com/scholar?hl=en&as_sdt=0%2C5&q=Yohannes%2BAyanaw%2BHabitu%2BPrevalence%2Band%2BFactors%2BAssociated%2Bwith%2BTeenage%2BPregnancy%2C%2BNortheast%2BEthiopia%2C%2B2017%3A%2BA%2BCross-Sectional%2BStudy&btnG) [DOI](https://doi.org/10.1155/2018/1714527)
316 8.	Poudel S, Upadhaya N, Khatri R, Ghimire P. Trends and factors associated with
317 pregnancies among adolescent women in Nepal: Pooled analysis of Nepal Demographic and

318	Health Surveys (2006, 2011 and 2016). PloS one. 2018;13(8):e0202107. [PubMed](https://pubmed.ncbi.nlm.nih.gov/30092087/) [DOI](https://doi.org/10.1371/journal.pone.0202107)

319 9.	Ministry of Health N, New ERA; and ICF, Nepal Demographic and Health Survey 2016
320 [Internet]. Kathmandu, Nepal: Ministry of Health, Nepal. 2017[cited 2023 Jan 2].. Available from:
321 <https://www.dhsprogram.com/pubs/pdf/fr336/fr336.pdf>
322 10.	Government of Nepal. National Demographic Census, Nepal 2011[Internet].National
323 Planning Commission: Kathmandu: Government of Nepal; 2012[cited 2023 Feb 20]. Avalable
324 from:	[https://unstats.un.org/unsd/demographic-social/census/documents/Nepal/Nepal-Census-](https://unstats.un.org/unsd/demographic-social/census/documents/Nepal/Nepal-Census-2011-Vol1.pdf)

325	[2011-Vol1.pdf](https://unstats.un.org/unsd/demographic-social/census/documents/Nepal/Nepal-Census-2011-Vol1.pdf)

326 11.	Poudel IA, Thulung B, Lopchan M. Consequences of teenage pregnancy among chepang
327 women in Korak VDC in Chitwan. Journal of Chitwan Medical College. 2016;6(2):21-6. [Google](https://scholar.google.com/scholar?hl=en&as_sdt=0%2C5&q=CONSEQUENCES%2BOF%2BTEENAGE%2BPREGNANCY%2BAMONG%2BCHEPANG%2BWOMEN%2BIN%2BKORAK%2BVDC%2BIN%2B%2BCHITWAN%2BI%2BAdhikari%2B%28Poudel%29%2B1%2B%2A%2C%2BB%2BThulung1%2B%2C%2BM%2BLopchan%2B1&btnG)
328 [Scholar](https://scholar.google.com/scholar?hl=en&as_sdt=0%2C5&q=CONSEQUENCES%2BOF%2BTEENAGE%2BPREGNANCY%2BAMONG%2BCHEPANG%2BWOMEN%2BIN%2BKORAK%2BVDC%2BIN%2B%2BCHITWAN%2BI%2BAdhikari%2B%28Poudel%29%2B1%2B%2A%2C%2BB%2BThulung1%2B%2C%2BM%2BLopchan%2B1&btnG) [Full text](https://www.nepjol.info/index.php/JCMC/article/view/16681/0)
329 12.	Panta P, Tripathi P, Sharma D, Amgain K. Knowledge and Practice of Family Planning
330 Methods among Married Women of Reproductive Age of Chepang Community in Benighat VDC
331 of Dhading District. Journal of Karnali Academy of Health Sciences. 2020;3(1):1-12. [Google](https://scholar.google.com/scholar?hl=en&as_sdt=0%2C5&q=Panta%2BP%2C%2BTripathi%2BP%2C%2BSharma%2BD%2C%2BAmgain%2BK.%2BKnowledge%2Band%2BPractice%2Bof%2BFamily%2BPlanning%2BMethods%2Bamong%2BMarried%2BWomen%2Bof%2BReproductive%2BAge%2Bof%2BChepang%2BCommunity%2Bin%2BBenighat%2BVDC%2Bof%2BDhading%2BDistrict.%2BJournal%2Bof%2BKarnali%2BAcademy%2Bof%2BHealth%2BSciences.%2B&btnG)
332 [Scholar](https://scholar.google.com/scholar?hl=en&as_sdt=0%2C5&q=Panta%2BP%2C%2BTripathi%2BP%2C%2BSharma%2BD%2C%2BAmgain%2BK.%2BKnowledge%2Band%2BPractice%2Bof%2BFamily%2BPlanning%2BMethods%2Bamong%2BMarried%2BWomen%2Bof%2BReproductive%2BAge%2Bof%2BChepang%2BCommunity%2Bin%2BBenighat%2BVDC%2Bof%2BDhading%2BDistrict.%2BJournal%2Bof%2BKarnali%2BAcademy%2Bof%2BHealth%2BSciences.%2B&btnG) [DOI](https://doi.org/10.3126/jkahs.v3i1.28654)
333 13.	Family Health Division, Department of Health Services, Ministry of Health, Government
334 of Nepal. Nepal Reproductive Health Strategy[Internet]. Kathmandu: Government of Nepal; 1998
335 [cited	2023	Feb	20]..	Available	from:	[https://km.mohp.gov.np/sites/default/files/2018-](https://km.mohp.gov.np/sites/default/files/2018-07/RH_Strategy.pdf)
336 [07/RH_Strategy.pdf](https://km.mohp.gov.np/sites/default/files/2018-07/RH_Strategy.pdf)
337 14. Shrestha DB, Budhathoki P, Shrestha O, Karki S, Thapa N, Dangal G, et al. Teenage Pregnancy
338 and Associated Risk Factors and Outcome in Nepal From 2000-2020: A Systematic Review and
339 Meta-Analysis. Kathmandu Univ Med J. 2022;[Full Text](http://www.kumj.com.np/issue/OnlineFirst/235-243.pdf) [Google Scholar](https://scholar.google.com/scholar?hl=en&as_sdt=0%2C5&q=Teenage%2BPregnancy%2Band%2BAssociated%2BRisk%2BFactors%2Band%2B%2BOutcome%2Bin%2BNepal%2BFrom%2B2000-2020%3A%2BA%2BSystematic%2B%2BReview%2Band%2BMeta-Analysis&btnG)
340 15. Bajracharya, A., S. R. Psaki and M. Sadiq. Child marriage, adolescent pregnancy and school
341 dropout in South Asia, report by the Population Council for the United Nations Children’s Fund
342 Regional	Office	for	South	Asia,	Kathmandu,	Nepal,	2019.Available	from:
343 UNICEF_ROSA_Child_marriage_adolescent_pregnancy_3May2019.pdf
344 16. Wodon Q, Montenegro C, Nguyen H, and Onagoruwa A. The cost of not educating girls.
345 Missed opportunities: the high cost of not educating girls. Children’s Investment Fund
346 Foundation,Global partnership for education, Mala Fund, World Bank Group. Jul 2018. Available
347 from:	[openknowledge.worldbank.org/server/api/core/bitstreams/01a423c9-8a5a-5a4f-bcf3-](https://openknowledge.worldbank.org/server/api/core/bitstreams/01a423c9-8a5a-5a4f-bcf3-2983fdb1b215/content)
348 [2983fdb1b215/content](https://openknowledge.worldbank.org/server/api/core/bitstreams/01a423c9-8a5a-5a4f-bcf3-2983fdb1b215/content)
349 17. Bongaarts J, Mensch BS, Blanc AK. Trends in the age at reproductive transitions in the
350 developing world: The role of education. Journal of demography. 2017. [Full Text](https://www.tandfonline.com/doi/full/10.1080/00324728.2017.1291986) [DOI](https://doi.org/10.1080/00324728.2017.1291986) [Google](https://scholar.google.com/scholar?hl=en&as_sdt=0%2C5&q=Trends%2Bin%2Bthe%2Bage%2Bat%2Breproductive%2Btransitions%2Bin%2Bthe%2Bdeveloping%2Bworld%3A%2BThe%2Brole%2Bof%2Beducation&btnG)
351 [Scholar](https://scholar.google.com/scholar?hl=en&as_sdt=0%2C5&q=Trends%2Bin%2Bthe%2Bage%2Bat%2Breproductive%2Btransitions%2Bin%2Bthe%2Bdeveloping%2Bworld%3A%2BThe%2Brole%2Bof%2Beducation&btnG)
352 18. McCleary-Sills J, Hanmer L, Parsons J, Klugman J. Child Marriage: A Critical Barrier to
353 Girls’ Schooling and Gender Equality in Education. The review of faith and international
354 affairs.2015. [Full Text](https://www.tandfonline.com/doi/full/10.1080/15570274.2015.1075755) [Google Scholar](https://scholar.google.com/scholar?hl=en&as_sdt=0%2C5&q=Child%2BMarriage%3A%2BA%2BCritical%2BBarrier%2Bto%2BGirls%E2%80%99%2BSchooling%2Band%2BGender%2BEquality%2Bin%2BEducation&btnG)
355 19. Samarakoon S, and Parinduri RA. Does Education Empower Women? Evidence from
356 Indonesia. World Development. 2015. 66; 428-42. [Google Scholar](https://scholar.google.com/scholar?hl=en&as_sdt=0%2C5&q=.%2BDoes%2BEducation%2BEmpower%2BWomen%3F%2BEvidence%2Bfrom%2BIndonesia%2B%28sciencedirectassets.com%29&btnG) [DOI](https://doi.org/10.1016/j.worlddev.2014.09.002)
357 20**.** 20 Advantages and Disadvantages of Joint Family in India[Internet]. Intellectual Gynai. 26 Nov
358 2022[cited	2023	March	18].	Available	from:	[https://intellectualgyani.com/advantages-](https://intellectualgyani.com/advantages-disadvantages-of-joint-family/)
359 [disadvantages-of-joint-family/](https://intellectualgyani.com/advantages-disadvantages-of-joint-family/)
360 21. Times of India .5 advantages of living in a joint family [Internet]. Times of India. 21 Jun 2023[
361 cited	20	Jul	2023].	Available	from:	[https://timesofindia.indiatimes.com/life-](https://timesofindia.indiatimes.com/life-style/relationships/love-sex/5-advantages-of-living-in-a-joint-family/articleshow/101050754.cms?from=mdr)
362 [style/relationships/love-sex/5-advantages-of-living-in-a-joint-](https://timesofindia.indiatimes.com/life-style/relationships/love-sex/5-advantages-of-living-in-a-joint-family/articleshow/101050754.cms?from=mdr)
363 [family/articleshow/101050754.cms?from=mdr](https://timesofindia.indiatimes.com/life-style/relationships/love-sex/5-advantages-of-living-in-a-joint-family/articleshow/101050754.cms?from=mdr)
364 22. Güneş PM. The role of maternal education in child health: Evidence from a compulsory
365 schooling law. [Economics of Education Review](https://www.sciencedirect.com/journal/economics-of-education-review).2015. 47;1-16. [Google Scholar](https://scholar.google.com/scholar?hl=en&as_sdt=0%2C5&q=The%2Brole%2Bof%2Bmaternal%2Beducation%2Bin%2Bchild%2Bhealth%3A%2BEvidence%2Bfrom%2Ba%2Bcompulsory%2Bschooling%2Blaw%2B&btnG)

SPSS data entry file

[Click here to access/download](https://www.editorialmanager.com/pone/download.aspx?id=33938451&guid=67fd37d9-dfdc-42af-bb7d-4a98dce046cd&scheme=1)

[**Supporting Information** Chepang Women 15th Aug.sav](https://www.editorialmanager.com/pone/download.aspx?id=33938451&guid=67fd37d9-dfdc-42af-bb7d-4a98dce046cd&scheme=1)
