# Supplementary material for: Factors associated with adolescent pregnancy among Chepang women and their health-seeking behavior in Ichchhakamana rural municipality of Chitwan district
Source: PLoS One. 2024 Mar 28;19(3):e0301261. doi: 10.1371/journal.pone.0301261 (PMC10977708; doi:10.1371/journal.pone.0301261)
Supplement: Attachment — Submitted filename: Response to Reviewers.docx [file pone.0301261.s009.docx]

**Journal Requirements:**

**Comment1:** Please review your reference list to ensure that it is complete and correct. If you have cited papers that have been retracted, please include the rationale for doing so in the manuscript text, or remove these references and replace them with relevant current references. Any changes to the reference list should be mentioned in the rebuttal letter that accompanies your revised manuscript. If you need to cite a retracted article, indicate the article’s retracted status in the References list and also include a citation and full reference for the retraction notice.

**Answer:** I have revised the references to correct some errors in links and also ensured that the references are correct. I have also removed all the hyperlinks and edited them according to journal guidelines. Additionally, I also checked if any of the cited references were retracted. But there were no retracted manuscripts.

**Additional Editor Comments:**

**Comment 2**: Correct Spelling in "Fisher Exact Test".

**Answer:** The correction has been made. (Lines 26, 153,242)

**Comment 3:** VIF values should be presented in result section.

**Answer:** The table of Collinearity Statistics has been added as a table in supplementary files (S2 table).

**Comment 4:** Why author did not consider non-response rate in sample size?

**Answer:** I apologize for the error. This portion was omitted by mistake in the last revision.

The calculated sample size was 216.8

Considering 10% non- response rate, total sample size = 216.8/0.9 = 240.8≈ 241

Therefore, the total sample size was 241.

However, Out of the 241 women chosen, a total of 217 participants responded, yielding a response rate of 90%. .

According to this information, necessary change has been made in the methodology (lines 120-121, 127-128) and result section of the manuscript (Lines 180).
