# Supplementary material for: Factors associated with adolescent pregnancy among Chepang women and their health-seeking behavior in Ichchhakamana rural municipality of Chitwan district
Source: PLoS One. 2024 Mar 28;19(3):e0301261. doi: 10.1371/journal.pone.0301261 (PMC10977708; doi:10.1371/journal.pone.0301261)
Supplement: Attachment — Submitted filename: Response to Reviewer 2024.docx [file pone.0301261.s010.docx]

**Response to editor:**

Comment 1: Website link of reference no 17 and 27 needs to be revised.

Answer 1: Thank you for the suggestion. The correction has been made. (lines 399 and 433-434)
